# Polyherbal formula (ASILACT®) induces Milk production in lactating rats through Upregulation of α-Lactalbumin and aquaporin expression

**DOI:** 10.1186/s12906-020-03152-7

**Published:** 2020-11-26

**Authors:** Mustofa, Fara Silvia Yuliani, Setyo Purwono, Ahmad Hamim Sadewa, Ema Damayanti, Didik Setyo Heriyanto

**Affiliations:** 1 Department of Pharmacology and Therapy, Faculty of Medicine, Public Health and Nursing, Universitas Gadjah Mada, Jl. Farmako, Sekip Utara, Yogyakarta, 55281 Indonesia; 2 Department of Biochemistry, Faculty of Medicine, Public Health and Nursing, Universitas Gadjah Mada, Jl. Farmako, Sekip Utara, Yogyakarta, 55281 Indonesia; 3 Research Division for Natural Product Technology, Indonesian Institute of Sciences, Jl. Jogja Wonosari KM 31.5, Gunungkidul, Yogyakarta, 55681 Indonesia; 4 Department of Anatomical Pathology, Faculty of Medicine, Public Health, and Nursing, Universitas Gadjah Mada, Jl. Farmako, Sekip Utara, Yogyakarta, 55281 Indonesia

**Keywords:** *Sauropus androgynous*, *Trigonella foenum-graceum*, *Moringa oleifera*, Galactagogue activity, α-Lactalbumin expression, Aquaporin expression

## Abstract

**Background:**

Polyherbal formula (PHF) contains extract of *Sauropus androgynous* (L.) Merr., *Trigonella foenum-graceum* L., and *Moringa oleifera* Lam. considered to induce galactagogue activity. This research aimed to evaluate the galactagogue activity of PHF and its effects on α-lactalbumin (LALBA) as well as aquaporin (AQP) gene expression at messenger ribonucleic acid (mRNA) levels in mammary glands of lactating rats.

**Methods:**

Thirty lactating Wistar rats were randomly divided into five groups (*n = 6*), each has 7 pups. Group I was treated orally with distilled water as negative control. Groups II, III, and IV were orally administered with PHF at 26.25, 52.5 and 105 mg/kg/day, respectively. Group V was treated with domperidone 2.7 mg/kg/day, orally as positive control. The treatment was performed at third day until fifteenth day of parturition. The observed parameters include the galactagogue activity indicating by milk yield of lactating rats, the pup weight changes and lactating rats body weight changes during lactating period, mRNA expression of LALBA and AQP using quantitative Real Time Polymerase Chain Reaction (qRT-PCR) and histopathological analysis of mammary glands at the end of treatment period.

**Result:**

The result showed that the PHF groups (52.5 and 105 mg/kg/day) and domperidone were significantly increased milk production of lactating rats (*p* < 0.05). The levels of mRNA expression of LALBA and AQPs were significantly upregulated by 105 mg/kg/day of PHF or 2.7 mg/kg of domperidone administration (*p* < 0.0001). Histopathological analysis of mammary glands shows that alveoli diameter was increase 14.59 and 19.33% at 105 mg/kg of PHF and 2.7 mg/kg of domperidone treatment, respectively.

**Conclusion:**

The study suggested that PHF has potentially to induce galactagogue activity on lactating period through upregulation of LALBA and AQP genes at the mRNA level.

**Supplementary Information:**

**Supplementary information** accompanies this paper at 10.1186/s12906-020-03152-7.

## Background

Low breast milk (hypogalactia) production in lactating mothers is among the most prominent reasons for discontinuation of breastfeeding in infants [1]. As breastfeeding is essential for the growth and development of infants, particularly in the first 6 months of life, the need to overcome this problem is crucial. Herbal galactagogues are widely used as traditional adjuvants to increase milk production in lactating mothers, especially among Indonesians. Herbal galactagogues are believed to have minimum side effects on the breastfeeding mother and on the baby relative to conventional galactagogues such as domperidone and metoclopramide [2].

*Sauropus androgynous* (L.) Merr. (Phyllanthaceae) leaf (locally known as *katuk*), *Trigonella foenum-graceum* L. (Fabaceae) (*klabet* seeds or fenugreek), and *Moringa oleifera* Lam. (Moringaceae) leaf (locally known as *kelor*) are well-known plants available throughout Indonesia and commonly used to increase milk production in lactating mothers. Previous research revealed that lactating mothers who consumed *S. androgynous* leaf extract produced 50.7% more milk than those who did not receive the extract [3]. *T. foenum-graceum*, widely recognized across the world as fenugreek, is also commonly used as a galactagogue. The seeds of this plant can significantly increase the proliferation of mammary glands in female mice [4, 5] . In addition, *M. oleifera* leaf is also used empirically as a galactagogue. Previous research has evidenced that milk volume in mothers increases after they receive *M. oleifera* leaf extract [6]. Considering the ability of these individual plants to increase milk production, a polyherbal formula (PHF) is expected to enhance their therapeutic action and to reduce the necessary concentration of individual plants, resulting in lower toxicity.

Nevertheless, the effect of a PHF on milk production on the level of the molecular mechanism remains unknown. α-Lactalbumin (LALBA) is a globular protein generated in epithelial cells of the mammary gland, which contributes to lactose synthesis, facilitating milk production and secretion [7, 8]. Combined with b-1,4-galactosyltransferase (B4GALTI), LALBA forms lactose synthase to convert glucose and galactose into lactose [7]. Aquaporins (AQP) are membrane proteins that play fundamental roles in facilitating water movement across cellular membranes and regulating water homeostatis [9]. The transport of water through the circulatory system and the movement of water to the mammary glands are crucial for milk synthesis and secretion [10]. AQP1 is mainly expressed in the capillaries, whereas AQP3 and AQP5 are mostly found in epithelial cells and ducts [11]. The individual plants in PHF have been found to be effective at increasing milk production. However, the effect of these plant combinations on milk production and its molecular mechanism remains unknown. Therefore, we aim to investigate the in vivo activity of a PHF containing *S. androgynus, T. foenum-graceum*, and *M. olerifera* on milk production, mRNA expression levels of LALBA, AQP1, AQP3, and AQP5 and the histopathology of mammary glands in lactating Wistar rats.

## Methods

### Animals

In vivo study using Lactating Wistar rats (175–200 g), procured from the Department of Pharmacology and Therapy, Faculty of Medicine, Public Health and Nursing, Universitas Gadjah Mada, Yogyakarta, Indonesia. Lactating Wistar rats were maintained under standard laboratory conditions (25 ± 2 °C and RH 60 ± 5%) with free access to food and water (ad libitum*).* The study was approved by the Medical and Health Research Ethics Committee (MHREC), the Faculty of Medicine, Public Health and Nursing at Universitas Gadjah Mada, Yogyakarta, Indonesia and ‘Dr. Sardjito’ General Hospital, Yogyakarta, Indonesia as the National Referral Hospital (KE/FK/0646/EC/2017).

### Polyherbal formula formulation (PHF)

The PHFs were prepared in capsule form by an herbal medicine industry (PT Swayasa Prakarsa, Yogyakarta, Indonesia) that met Good Manufacturing Practice standards for traditional medicine. Products containing these three plant extracts have now been produced by local companies and are available in the market under the trademark ASILACT® and are currently being registered with the Indonesian Food and Drug Administration. These herbals were extracted using aqueous solvent. Each capsule (500 mg) contained the following ingredients: *S. androgynous Folium* (300 mg)*, T. foenum-graceum* (150 mg)*,* and *M. oleifera Folium* (150 mg) based on preparation available in market*.* Product standardization refers to the parameters of a maximum moisture content, heavy metals (Pb and Cd) content, the total of yeast and fungi, the total plate number, the pathogenic microbes content, and the disintegration time which refers to the quality requirements of traditional medicines set by the Indonesian Food and Drug Administration.

### Galactagogue activity of the polyherbal formulation

Thirty lactating rats each of which have seven pups were divided into five groups. The negative control group of rats (Group I) were treated with orally administered distilled water; Groups II, III, and IV were treated with orally administered 26.25, 52.5, and 105 mg/kg/day of PHF, respectively. These selected doses based on pervious study [12]. As a positive control, Group V was treated with orally administered 2.7 mg/kg/day of domperidone (DOM® suspension, PT LAPI Laboratories, Serang, Indonesia). All the samples were administered to the mother rat groups from the 3rd to the 15th day of parturition. Every day during the study period, 13 h after the treatment of the mother rats, all pups were weighed (w1) and subsequently isolated from their mothers for 4 h. At the end of 4 h, the pups were weighed (w2), returned to their mothers and allowed to feed for 1 h and were weighed (w3) again. The daily milk yield at 18 h was corrected for weight loss due to metabolic processes in the pup (respiration, urination, and defecation) during suckling. The value used was (w2 − w1)/4. This value was multiplied by the number of suckling hours per day and added to the daily suckling gain. The pups’ daily weight gain was calculated from their weight at w2. The weights of mother rats were measured daily, and the differences in weight between the 3rd and 15th days of parturition were calculated. This research report uses the ARRIVE Guidelines [13] which display a checklist as Additional file 1.

### Histopathology of the mammary gland

On the 16th day, all the mother rats (30/30) were sacrificed using double dose of cocktail dosage (0.150 mL/100 g body weight injected intraperitoneal). The composition of 10 mL cocktail dosage for euthanasia procedures were 2.5 mL of ketamine (Holland) 100 mg/mL; 2.5 mL of xylazine (Xyla®, Holland) 20 mg/mL; 1.0 mL of acepromazine (Holland Interchemie) 10 mg/mL; 4 mL sterile water (Ikapharmindo) for injection. Mammary glands tissues were fixed with 10% neutral buffer formalin and embedded in paraffin. Sections (5 μm) was used for hematoxylin and eosin (H&E) staining. Microscopic findings were captured for analysis using QuPath version 0.2.0-m2 with 400× magnification.

### Quantitative real-time PCR (qRT-PCR)

Expression of LALBA and AQPs by qRT-PCR was performed on RNA extracted from formalin-fixed paraffin-embedded tissues using the RibospinTM II RNA 116 Purification Kit (Cat. No. 314–150) according to the manufacturer’s instructions. qRT-PCR was performed on One-Step qRT-PCR with KAPA SYBR FAST Universal according to the manufacturer’s instructions. The PCR forward primers used here were.

GADPH (5′-GCA TCC TGG GCT ACA CTG AG-3′), LALBA (5′-AAG TAG TGA GTT CCC CGA GTC-3′), AQP1 (5′-TTA ACC CTG CTC GGT CCT TT-3′), AQP3 (5′-TTA ACC CTG CTC GGT CCT TT-3′), and AQP5 (5′-TGG AGC AGG CAT CCT GTA CT-3′). The reverse primers used included GAPDH (5′-TCC ACC CTG TTG CTG TA-3′), LALBA (5′-GGC TTT CCA GTA GTC GAT TCC TT-3′), AQP1 (5′-TTC ATC TCC ACC CTG GAG TT-3′), AQP3 (5′-GGA GCG TTT TTA GCC CGA GA-3′), and AQP5 (5′-CGT GGA GAA GAT GCA GA-3′). An individual reaction was carried out using the Bioneer ExicyclerTM96 Real-Time Quantitative 122 Thermal Block with reverse transcription at 42 °C for 5 min, followed by enzymatic activation at 95 °C for 3 min, denaturation for 1–3 s at 95 °C, and elongation for up to 20 s at 60 °C. Gene expression levels were calculated based on the cycle threshold (Ct) value using the following formula:

$$ Fold\ change={2}^{\left(-\varDelta \varDelta CT\right)} $$

$$ \varDelta CT=\left( CT\  LALBA\ or\  AQP1\  or\  AQP3\  or\  AQP5\right)- CT\  GADPH\ \left( internal\ control\right) $$

$$ \varDelta \varDelta CT=\varDelta CT\ (treatment)-\varDelta CT\ (control) $$

### Statistical analysis

Data were analyzed using PRISM 8 for macOS Version 8.4.2 (464). Data was expressed as the mean ± SD. Differences in gene expression among multiple group comparisons were first assessed by a one-way analysis of variance followed by Dunnett’s multiple comparison test. Significant differences were defined as those with *P* values smaller than 0.05.

## Results

### Galactagogue activity of the PHF

**Fig. 1:**
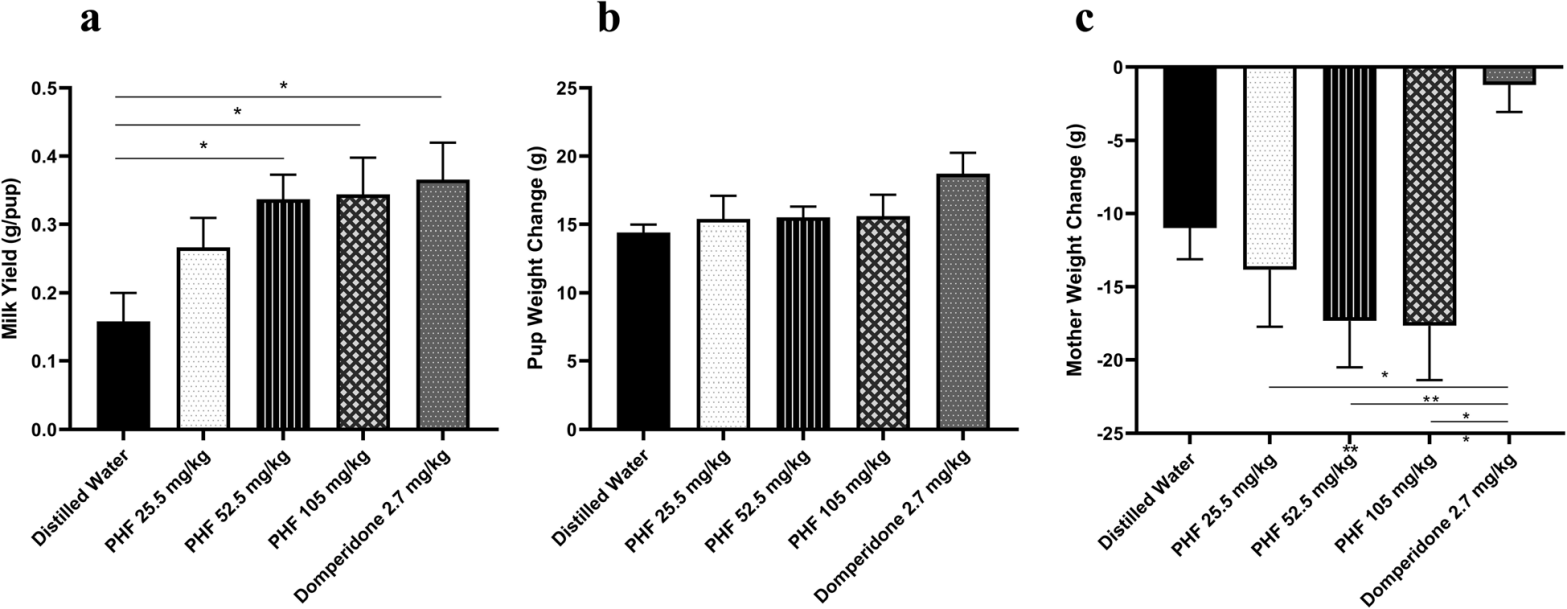
**a** Milk yield of lactating rats, **b** changes in pup weight (no significant difference),and **c** lactating Wistar rat body weight during lactating periods. The polyherbal formula (PHF) containing *Sauropus androgynous Folium* (300 mg), *Trigonella foenum-graceum* (150 mg), and *Moringa oleifera Folium* (150 mg)*.* The negative control group of rats was orally administered distilled water; treated groups were orally administered 26.25, 52.5, and 105 mg/kg/day of PHF, respectively. As a positive control was treated orally with 2.7 mg/kg/day of domperidone. All the PHFs were administered to the mother rat groups from the 3rd to the 15th day of parturition. Values are expressed as mean ± SEM (*p < 0.05, **p < 0.01)

Milk production in the groups treated with the PHF and domperidone was higher than those in the control group (Fig. 1 a). The mean ± SEM milk yield for the negative control group (distilled water) was 0.16 ± 0.42 g/pup per day. The groups to which PHF was administered at 26.25, 52.5, and 105 mg/kg were 0.28 ± 0.04, 0.34 ± 0.04, and 0.34 ± 0.05 g/pup per day, respectively, while the group that was treated with the positive control (domperidone 2.7 mg/kg) was 0.37 ± 0.054 g/pup per day. Significant differences were observed in the two highest doses of PHF and domperidone compared with the control group (*p* < 0.05).

Figure 1 b displays the increase in pup weight among different treatments. The mean pup weight change in the group treated with distilled water was 26.25, 52.5, and 105 mg/kg/day, and in the domperidone group 2.7 mg/kg it was 14.42 ± 0.56, 15.40 ± 1.69, 15.52 ± 0.78, 15.60 ± 1.56, and 18.71 ± 1.54 g, respectively. The negative control group (distilled water) showed the lowest growth rates among all groups. The rats treated with PHF and domperidone indicated no significant difference in pup weight gain; nevertheless, domperidone showed the highest pup weight gain of all groups.

All groups displayed a reduction in the body weights of the mother rats (Fig. 1 c). The mean body weight reduction in mother rats treated with distilled water was 26.25 mg/kg, 52.5 mg/kg, and 105 mg/kg, and in domperidone 2.7 mg/kg it was − 11 ± 2.09, − 13.83 ± 3.90, 17.33 ± 3.17, − 17.67 ± 3.71, and − 1.2 ± 1.86 g, respectively. The highest reduction in body weight was 105 mg/kg of PHF treatment, and the lowest reduction in body weight was with domperidone treatment. There were no significant differences between the PHF treatment group and the negative control (distilled water). Nevertheless, the PHF treatment group showed a significantly lower body weight reduction than the positive control (domperidone).

### Histopathology of the mammary gland

**Fig. 2:**
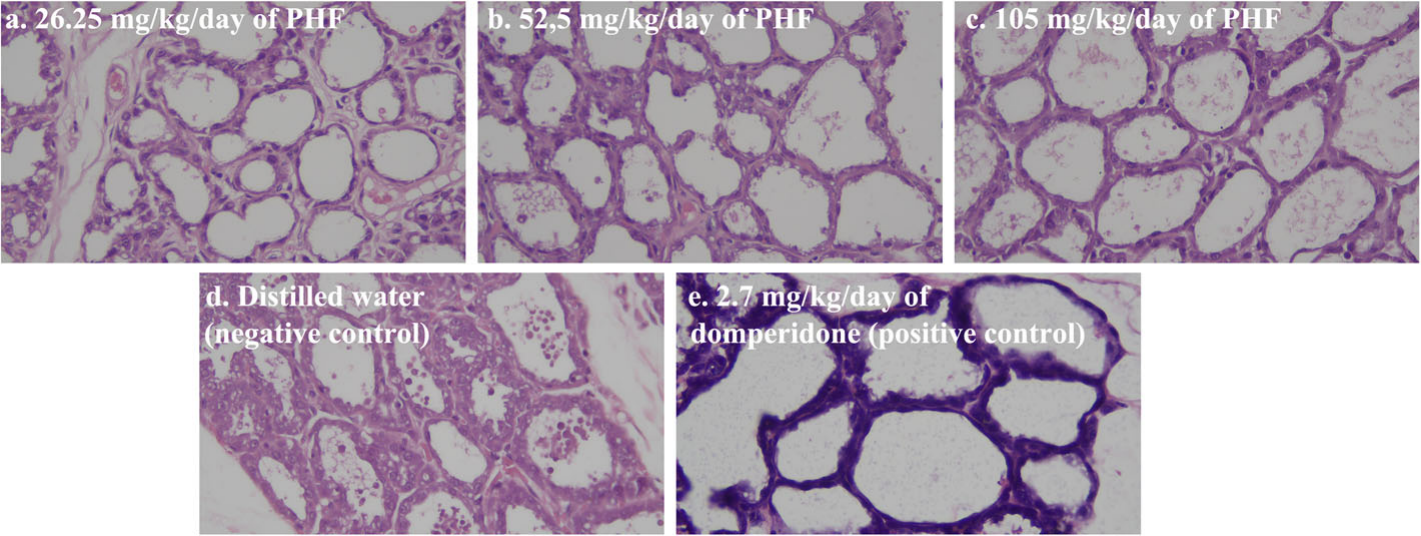
The effect of PHF administration on the alveolar diameter in the mammary glands of lactating Wistar rats (Hematoxylin Eosin stain, 400x magnification) at the end of treatment. The polyherbal formula (PHF) containing *Sauropus androgynous Folium* (300 mg), *Trigonella foenum-graceum* (150 mg), and *Moringa oleifera Folium* (150 mg)*.* All the PHFs were administered to the mother rat groups from 3rd to the 15th day of parturition

Histopathological examination (Fig. 2) of mother rats treated with 105 mg/kg of PHF administration and 2.7 mg/kg of the positive control (domperidone administration) led to an increase in the diameter of alveoli by 14.59 and 19.33%, respectively.

### Polyherbal formulation increased the mRNA expression of a-Lactalbumin and Aquaporins

**Fig. 3:**
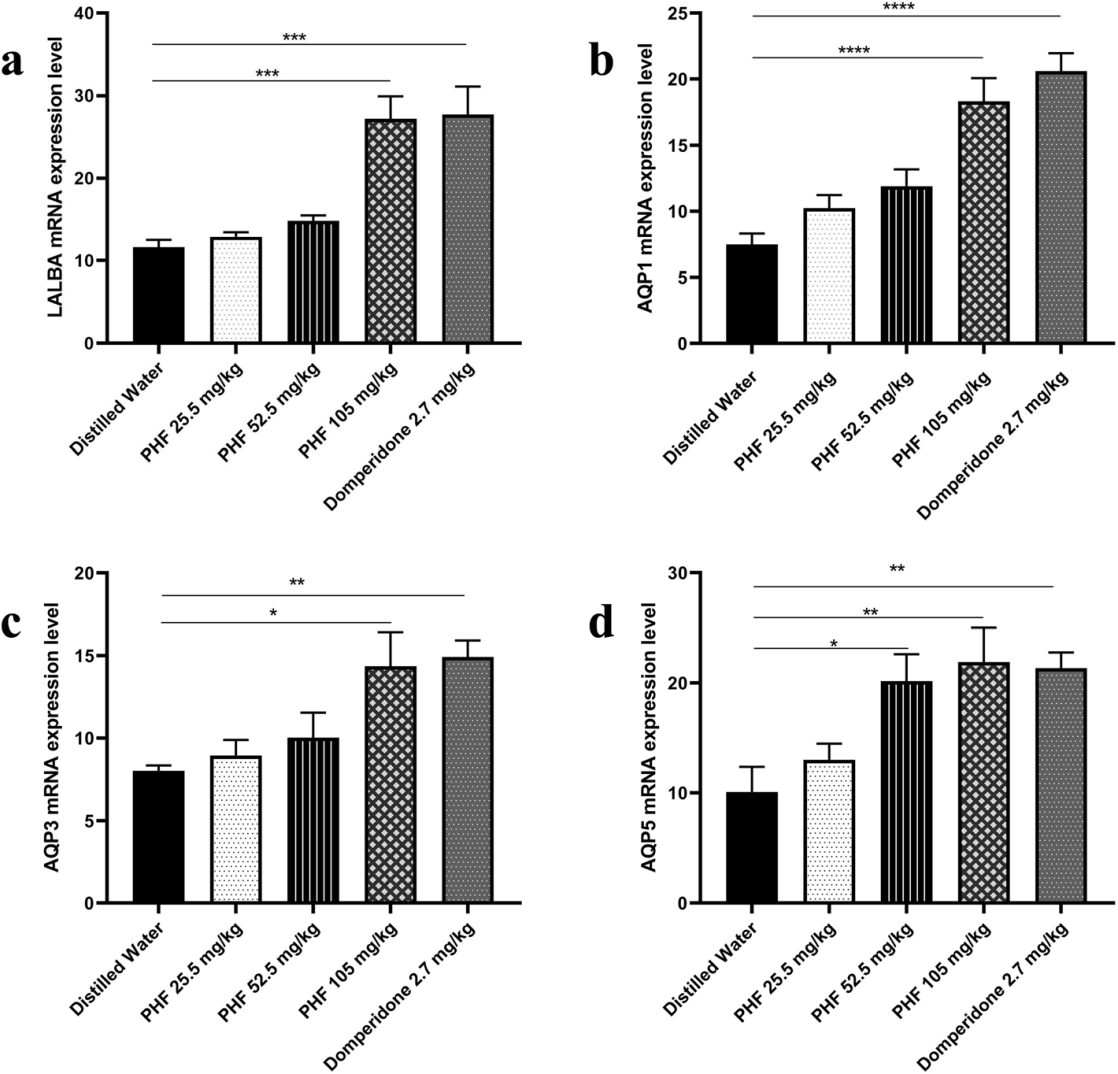
**a** The mRNA expression level of α-lactalbumin (LALBA), **b** Aquaporin 1 (AQP1), **c** Aquaporin 3 (AQP3), and **d** Aquaporin 5 (AQP5) in mammary gland tissues of lactating Wistar rats using quantitative Real-Time Polymerase Chain Reaction (qRT-PCR) analysis at the end of treatment. The polyherbal formula (PHF) containing *Sauropus androgynous Folium* (300 mg), *Trigonella foenum-graceum* (150 mg), and *Moringa oleifera Folium* (150 mg)*.* The negative control group of rats was orally administered distilled water; treated groups were orally administered 26.25, 52.5, and 105 mg/kg/day of PHF, respectively. A positive control group was treated orally with 2.7 mg/kg/day of domperidone. All the PHFs were administered to the mother rat groups from the 3rd to the 15th day of parturition. Values are expressed as mean ± SEM. (*p < 0.05, **p < 0.01, ***p < 0.001, ****p < 0.0001)

Quantitative RT-PCR analysis was performed to assess the mRNA expression of LALBA, AQP1, AQP2, and AQP 3. The incremental dose of PHF increased the expression of LALBA in mammary glands of mother rats, as depicted in Fig. 3. Compared with the negative control (distilled water), the expression of LALBA was significantly higher in 105 mg/kg of PHF administration (*p* < 0.0001) and 2.7 mg/kg of domperidone administration (positive control) (*p* < 0.0001), while domperidone as the reference drug showed the highest expression level of all groups. The mRNA expression levels of AQP1, AQP3, and AQP5 was more pronounced in the mammary glands of mother rats treated with 105 mg/kg of PHF and 2.7 mg/kg of domperidone (*p* < 0.0001) compared with the negative control (Fig. 3).

## Discussion

This study set out to assess the effect of PHF containing *S. androgynus, T. foenum-graceu,* and *M. oleifera* on milk production and the mRNA expression levels of LALBA, AQP1, AQP3, and AQP5 in the mammary glands of lactating rats. Measurement of pup weight and weight gain in rats has been used in several studies [11, 14–16]. With respect to the first purpose of this study, it was discovered that PHF possessed lactogenic activity and contributed to changes in several factors involved in lactating rats. Milk yield appeared to be significantly higher in lactating rats treated with PHF (at dose levels of 52.5 mg/kg and 105 mg/kg) and the reference drug domperidone (2.7 mg/kg) as a positive control. The qRT-PCR assay demonstrated that the mRNA expression levels of LALBA, AQP1, AQP3, and AQP5 increased in a dose-dependent manner, with 105 mg/kg of PHF administration and 2.7 mg/kg of domperidone administration (positive control) being significantly higher than the negative control (distilled water). We used domperidone as a reference as the drug has been proven to increase milk production by blocking dopamine activities, hence stimulating prolactin production.

The compounds of each plant in PHF might contribute to the upregulation of mRNA expression in LALBA, AQP1, AQP3, and AQP5, resulting in increased milk production. α- Lactalbumin is a globular protein generated in the epithelial cells of mammary glands and plays an important role during milk production. Combined with b-1,4-galactosyltransferase (B4GALTI), LALBA forms lactose synthase to convert glucose and galactose into lactose [7]. Lactose synthetase (LALBA and B4GALT1) and glucose transport (GLUT1) contribute to increased lactose synthesis in sows [17]. α-Lactalbumin and the activity of lactose synthase are enhanced by prolactin mediated by STAT5a [18, 19]. The upregulation of APQs in the mammary glands of rats treated with PHF in this study supports previous findings suggesting the involvement of these proteins in lactation. The importance of AQPs during lactation has been noted by Benoit et al [20], who suggested that the reduction of water movement into milk glands was due to AQP impairment in the Tsetse fly.

Immunohistochemistry techniques have confirmed the presence of AQP1, AQP3, and AQP5 water channels in mammalian and human mammary glands. It shown that AQP1 is responsible for transferring water from the blood to the interstitial space, while AQP3 transfers water and/or glycerol from interstitial fluid to the cytoplasm [[21]. Furthermore, AQP5 transports water passively across the membrane, depending on the osmotic gradient [22]. The effect of an herbal galactagogue on AQPs has also been demonstrated by Liu et al. [11]. They discovered that a herbal galactagogue mixture was able to increase AQP3 and AQP5 protein expression, but not that of AQP1, in mammary glands of lactating rats. This result differed slightly from our findings, which demonstrated that AQP1 was also upregulated in the PHF and domperidone group. These findings give an indication that the contents of PHF and domperidone in our study also had an effect on the capillaries of mammary glands, where AQP1 is abundantly expressed.

The upregulation of LALBA and AQP mRNA expression in PHF-treated rats might be influenced by the stimulation of lactogenic hormones. Shao et al. demonstrated that the expression of LALBA mRNA in bovine mammary epithelial cells increased several hundred-fold after prolactin stimulation combined with insulin, hydrocortisone, and estradiol [23]. Moreover, prolactin and estrogen have been recognized to result in upregulation of AQP1, AQP3, and AQP5 expression [24–26]. The individual plants in PHF are known for their ability to increase milk production in animal models and in humans by stimulating lactating hormones [27–30]. A previous study suggested that levels of prolactin and oxytocin were higher in lactating BALB/C mice supplemented with *S. androgynus* leaf extract [28]. The presence of papaverine, the secondary metabolite in *S. androgynus*, may contribute to the increase in milk supply. Papaverin impedes the activity of phosphodiesterase, causing cAMP accumulation. A high level of cAMP will relax the smooth muscles surrounding the blood vessels [31]. Moreover, papaverine is also a vasodilator. When the vessels dilate, blood flow increases, which leads to the smooth circulation of prolactin and oxytocin through the bloodstream. *Saoropus androgynus* leaves also contain a sterol that plays a role in hormone precursors and increasing estrogen production. In other study, phytochemical analyses of *S. androgynus leaves* identified high content of fatty acids, flavonoids, and polyphenols as the major bioactive components [32]. The sterol compound which estrogen precursors is also found in *M. oleifera* leaves as stigmasterol, sitosterol, and kampesterol [28]. The bioactive constituent analysis in *M. oleifera* also found glucosinolates, flavonoids and phenolic acids, carotenoids, tocopherols, polyunsaturated fatty acids (PUFAs), highly bioavailable minerals, and folate. In the leaves, the amount of quercetin and kaempferol was found to be in the range of 0.07–1.26 and 0.05–0.67%, respectively [33]. A previous clinical trial demonstrated that *T. foenum-graceum* seed increased lactogenesis and prolactin levels in the early stages of lactation [34]. The phytochemical contents of *T. foenum-graceum* seed, including isoflavones, alkaloids, polyphenols, tannins, and saponins, enhanced prolactin release, which in turn stimulated milk ejection, protein levels, and lactation [35]. The oils extract of *T. foenum-graceum* were rich in unsaturated fatty acids (UFA) (oleic acid, linoleic acid, and linolenic acid making up from 83.19 to 83.33% of total fatty acids) and low in saturated fatty acids (SFA) (primarily palmitic acid, making up from 9.94 to 10.00% of total fatty acids) [36]. An estrogen-like effect or phytoestrogen and diosgenin (a type of steroidal sapogenin) in fenugreek or *T. foenum-graceum* have been considered as responsible compounds for the galactagogue effect in this herb [4]. *Moringa oleifera* also possesses the ability to increase prolactin levels and milk production in mothers supplemented with *M. oleifera* compared with the control group [27, 29].

In addition, our findings revealed that the PHF-treated rat group experienced weight loss. Other studies exhibited that *Sauropus androgynus* leaves demonstrate an ability to reduce body weight [37, 38]. It was suggested that *S. androgynus* leaves reduced fat accumulation in broiler chickens [39]. In contrast, it has been reported that domperidone contributes to maternal weight gain [40]. Nevertheless, lactating rats treated with domperidone in our study demonstrated weight loss; however, the reduction was considerably smaller than that in the control (distilled water). Pup weight in the PHF groups increased in a dose-dependent manner; however, no significant difference from the control was observed. This finding was consistent with a previous study, which suggested that pup weight gain was not consistent with increased milk production [17].

## Conclusion

The Polyherbal Formula (PHF) containing *Sauropus androgynous* (L.) Merr. leaves extract, *Trigonella foenum-graceum* L. seed extract, and *Moringa oleifera* Lam. leaves extract possess significant galactagogue activity. PHF administration in lactating rats induced milk production by upregulating the LALBA, AQP1, AQP3, and AQP5 genes in mammary glands. Evaluation of PHF toxicity in a preclinical and clinical study is expected in order to develop this preparation to be phytochemical medicine.

## Supplementary Information

**Additional file 1.** The ARRIVE Guidelines Checklist. Animal Research: Reporting In Vivo Experiments.

## Data Availability

All data analysed during this study are included in this published article. The datasets generated during this study are not publicly available but are available from the corresponding author on reasonable request.

## Abbreviations

PHF: Polyherbal formula
mRNA: Messenger ribonucleic acid
LALBA: Α-lactalbumin
AQP: Aquaporin
qRT-PCR: Real time polymerase chain reaction
B4GALTI: B-1,4-galactosyltransferase
